# Burden of arrhythmia in hospitalized HIV patients

**DOI:** 10.1002/clc.23506

**Published:** 2020-12-09

**Authors:** Anas A. Abudan, Vaibhav R. Vaidya, Byomesh Tripathi, Peter A. Noseworthy, Daniel C. DeSimone, Alexander Egbe, Shilpkumar Arora, Haarini Sridhar, Christopher V. DeSimone, Siva Mulpuru, Abhishek J. Deshmukh

**Affiliations:** ^1^ Division of Cardiovascular Diseases Mayo Clinic Rochester Minnesota United States; ^2^ Department of Internal Medicine University of Kansas School of Medicine Kansas City Kansas United States; ^3^ Department of Medicine Mount Sinai St Luke's‐Roosevelt Hospital New York New York United States; ^4^ Division of Infectious Diseases Mayo Clinic Rochester Minnesota USA; ^5^ University of California Berkeley California United States

**Keywords:** arrhythmia, atrial fibrillation, frequency, human immunodeficiency virus (HIV)

## Abstract

**Background:**

The improved life expectancy observed in patients living with human immunodeficiency virus (HIV) infection has made age‐related cardiovascular complications, including arrhythmias, a growing health concern.

**Hypothesis:**

We describe the temporal trends in frequency of various arrhythmias and assess impact of arrhythmias on hospitalized HIV patients using the Nationwide Inpatient Sample (NIS).

**Methods:**

Data on HIV‐related hospitalizations from 2005 to 2014 were obtained from the NIS database using International Classification of Diseases, 9th Revision (ICD‐9) codes. Data was further subclassified into hospitalizations with associated arrhythmias and those without. Baseline demographics and comorbidities were determined. Outcomes including in‐hospital mortality, cost of care, and length of stay were extracted. SAS 9.4 (SAS Institute Inc., Cary, NC) was utilized for analysis. A multivariable analysis was performed to identify predictors of arrhythmias among hospitalized HIV patients.

**Results:**

Among 2 370 751 HIV‐related hospitalizations identified, the overall frequency of any arrhythmia was 3.01%. Atrial fibrillation (AF) was the most frequent arrhythmia (2110 per 100 000). The overall frequency of arrhythmias increased over time by 108%, primarily due to a 132% increase in AF. Arrhythmias are more frequent among older males, lowest income quartile, and nonelective admissions. Patients with arrhythmias had a higher in‐hospital mortality rate (9.6%). In‐hospital mortality among patients with arrhythmias decreased over time by 43.8%. The cost of care and length of stay associated with arrhythmia‐related hospitalizations were mostly unchanged.

**Conclusions:**

Arrhythmias are associated with significant morbidity and mortality in hospitalized HIV patients. AF is the most frequent arrhythmia in hospitalized HIV patients.

## 1 INTRODUCTION

According to the Center for Disease Control and Prevention (CDC), there were 39 782 incident cases of human immunodeficiency virus (HIV) infection in 2016 in the United States.^1^ Access to effective antiretroviral therapy (ART) has substantially reduced HIV mortality, acquired immune deficiency syndrome (AIDS), and AIDS‐related hospitalizations. This has led to an overall improved life expectancy in the HIV patient population.^2^ However, in the aging HIV‐infected population, cardiovascular complications such as hypertension, coronary artery disease (CAD) and heart failure have become a growing health concern.^3^, ^4^, ^5^, ^6^ Other contributors such as the HIV infection itself, immune dysfunction, chronic inflammation, ART exposure and toxicity are also implicated in heart disease and can lead to complications such as myocardial infarction and cardiomyopathy.^7^, ^8^ In addition, arrhythmias are also important contributors to cardiovascular morbidity and mortality in patients with HIV.^9^, ^10^ The temporal trends of the frequency and outcome of arrhythmias in patients with HIV have not been adequately described.

## 2 METHODS

The primary objective of this study was to describe the temporal trends in the frequency of arrhythmias among hospitalized HIV patients. Secondary objectives included identifying comorbidities associated with arrhythmias in this cohort as well as determine the outcomes associated with arrhythmias including in‐hospital mortality, length of stay, and cost of care.

### 2.1 Data source

The data were obtained from the Nationwide Inpatient Sample (NIS) data set from 2005 to 2014.^11^ The NIS is a nationally representative survey of hospitalizations conducted by the Healthcare Cost and Utilization Project in collaboration with the participating states. It is the largest inpatient data set in the United States and includes a sample of US community hospitals that approximates 20% of all US community hospitals.^12^ No institutional review board approval was sought because of the publicly available de‐identified data set used in this research.

### 2.2 Study population

Our target population consisted of HIV‐related hospitalizations from January 1, 2005 to December 21, 2014. We included hospital admissions with a diagnosis of HIV infection in primary and secondary diagnostic field during our study period. We subclassified this group into hospitalizations with associated cardiac arrhythmia and those without arrhythmia for trend analysis using ICD codes for cardiac arrhythmias such as ventricular tachycardia (VT), ventricular fibrillation (VF), supraventricular tachycardia (SVT), AF, and atrial flutter (AFL). Interventions such as ICD implantation, use of vasopressors, cardiac catheterization, endotracheal intubation, and CPR were identified.

### 2.3 Definition of variables

We used NIS variables to identify patient level and hospital level variables. We divided age into 4 subgroups: 18 to 49 years of age, 50 to 64 years of age, 65 to 79 years of age, and 80 years of age and older. We defined the severity of comorbid conditions by using the Deyo modification of the Charlson Comorbidity Index. Co‐morbidities associated with hospitalization for HIV infection were identified using AHRQ comorbidity measures, that is, by using ICD‐9‐CM diagnoses and the Diagnosis Related Group (DRG) in effect on the discharge date.^13^

### 2.4 In hospital mortality, cost, and length of stay

In‐hospital mortality was defined as death from any cause during the same hospital stay. LOS was already provided by the Healthcare Cost and Utilization Project (HCUP) for each entry. The HCUP NIS contains data on total charges for each hospital in the databases, which represents the amount that hospitals billed for services. To calculate estimated cost of hospitalizations, the NIS data were merged with Cost to Charge Ratios (CCR) available from HCUP.^14^, ^15^ Using the merged data elements from the CCR files and the total charges reported in the NIS database, we converted the hospital total charge data to cost estimates by simply multiplying total charges with the appropriate CCR. These costs are essentially standardized, can be measured across hospitals, and are used for the remainder of this report.

### 2.5 Statistical analysis

The statistical software package SAS 9.4 (SAS Institute Inc., Cary, NC) was utilized for the analyses. Since NIS represents a 20% stratified random sample of US hospitals, analyses were performed using hospital‐level discharge weights to obtain national estimates of arrhythmia associated with HIV hospitalizations. For categorical variables, the modified chi‐squared test of trend for proportions (Cochrane Armitage test) was used. For continuous variable, simple linear regression was used. Multivariable models for predictors of arrhythmia were performed with independent variables which were either clinically significant or statistically significant in univariate model. Multivariate model included patient‐level variables such as age and gender; year of admission, admission type (elective vs nonelective), co‐morbidities such as diabetes mellitus, chronic lung disease, peripheral vascular disease etc.; hospital course requiring ICD implantation, use of vasopressors, cardiac catheterization, endotracheal intubation and CPR. A *P*‐value <.05 was deemed statistically significant.

## 3 RESULTS

We identified 2 370 751 HIV‐related hospitalizations from January 1, 2005 through December 31, 2014. The frequency of all variables is per 100 000 hospitalizations, unless specifically mentioned.

### 3.1 Frequency of arrhythmias

Among 2 370 751 HIV‐related hospitalizations, 71 285 hospitalizations had either a primary or secondary arrhythmia‐related diagnosis. This amounts to a 3.01% frequency of arrhythmia‐related diagnoses in HIV related hospitalization (Table 1). The frequency of specific arrhythmias (per 100 000 hospitalizations) in the study was as follows: 2110 AF, 560 VT, 420 AFL, 170 SVT, and 130 VF.

**TABLE 1:** Comparison of baseline characteristics and course of hospitalization between HIV patients with arrhythmias and without arrhythmias from 2005 through 2014

	Patients with arrhythmia	Patients without arrhythmia	Overall	*P* value
Primary admission	71 285 (3.01%)	2 299 466	2 370 751	
**Patient level variables**				
**Age**				<.0001
18‐49	30.42	62.2	61.24	
50‐64	49.25	33.38	33.85	
65‐79	18.47	4.21	4.64	
≥80	1.87	0.22	0.27	
**Gender**				<.0001
Male	76.42	65.73	66.05	
Female	23.58	34.27	33.95	
**Deyo/Charlson Score**				<.0001
<2	18.36	27.58	27.3	
>=2	81.64	72.42	72.7	
**Hospital course**				
Use of vasopressor	1.3	0.24	0.28	<.0001
Cardiac catheterization	7.17	1.54	1.71	<.0001
Endotracheal intubation	12.74	3.7	3.97	<.0001
CPR	3.4	0.41	0.5	<.0001
Cardiogenic shock	1.9	0.12	0.17	<.0001
Cardiac arrest	4.09	0.42	0.53	<.0001
**Median household income category for patient's zip code**				<.0001
1. 0‐25th percentile	44.85	49.39	49.25	
2. 26‐50th percentile	22.9	23.05	23.05	
3. 51‐75th percentile	19.16	17.03	17.09	
4. 76‐100th percentile	13.09	10.54	10.61	
**Primary payer**				<.0001
Medicare/Medicaid	74.72	70.13	70.27	
Private including HMO	17.36	15.7	15.75	
Self‐pay/no charge/other	7.8	13.95	13.76	
Missing	0.12	0.22	0.22	
**Admission type**				<.0001
Nonelective	90.77	88.28	88.36	
Elective	9.23	11.72	11.64	
**Admission day**				<.0001
Weekdays	78.14	78.85	78.83	
Weekend	21.86	21.15	21.17	
**Hospital characteristics**				
**Hospital bed size**				<.0001
Small	8.64	9.38	9.36	
Medium	24.47	24.3	24.31	
Large	66.89	66.32	66.34	
**Hospital teaching status**				<.0001
Nonteaching	30.75	29.33	29.38	
Teaching	69.25	70.67	70.62	
**Discharge**				<.0001
Home	68.33	78.78	78.46	
Facility	19.04	12.76	12.95	
In hospital mortality	9.6	2.84	3.04	<.0001
Length of stay during index admission in days (median, IQR)	5(3‐10)	4 (2‐7)	4 (2‐7)	<.0001
Cost of care (median, IQR)	12 210 (6248‐26 167)	7577 (4401‐14 083)	7665 (4435‐14 336)	<.0001

Abbreviations: CPR, cardiopulmonary resuscitation; HMO, Health Maintenance Organization; IQR, interquartile range.

**FIGURE 1:**
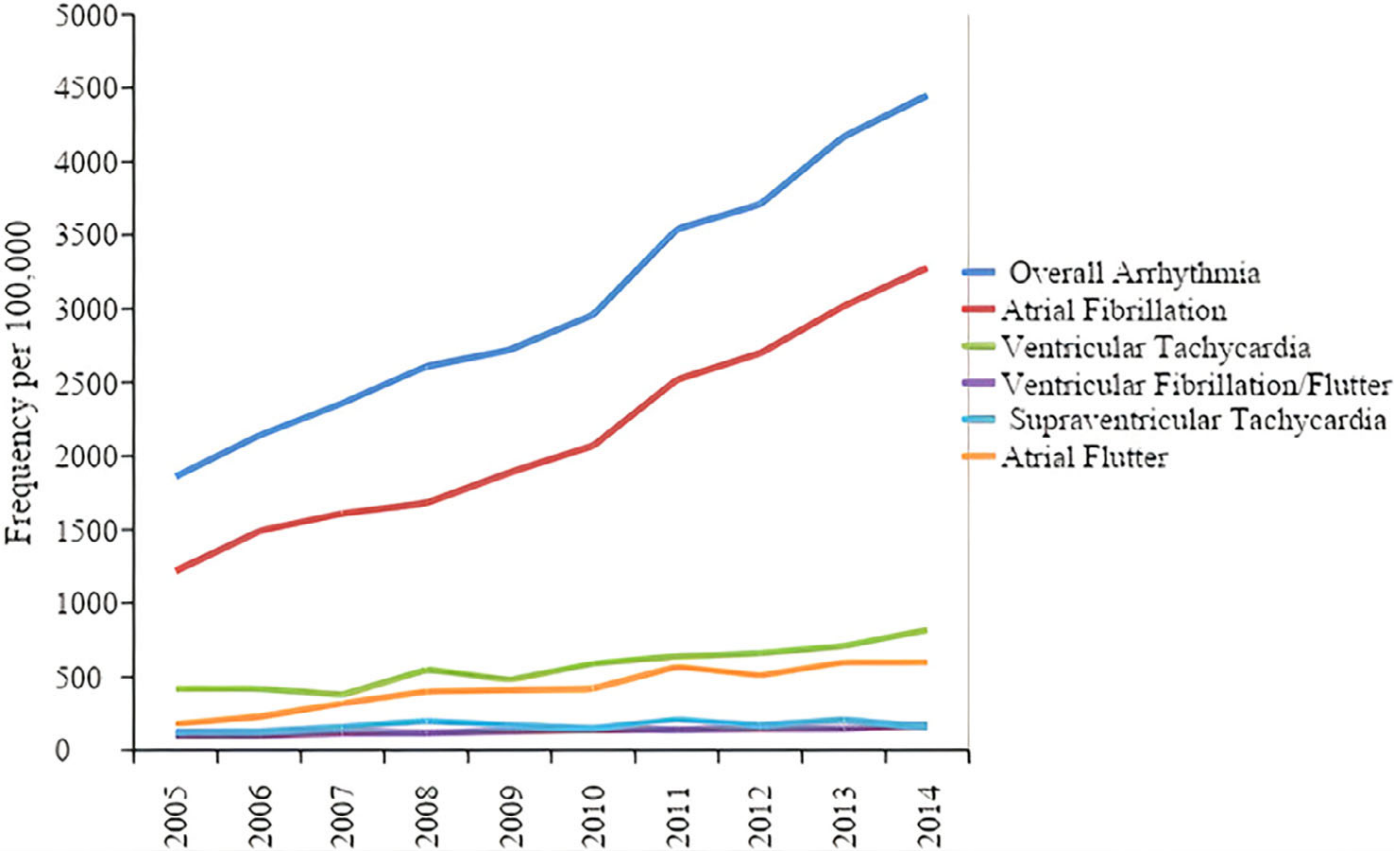
Frequency of arrhythmia‐related hospitalizations by arrhythmia type for the entire study period

From 2005 through 2014, the frequency of arrhythmia among HIV‐related hospitalizations increased by 108% (from 4412 in 2005 to 9435 in 2014). This increase in arrhythmia was primarily the result of the increased frequency of AF (132.5% increase, from 2898 patients in 2005 to 6955 in 2014), followed by VT (72.1% increase, from 985 in 2005 to 1735 in 2014) and AFL (192.7% increase, from 427 in 2005 to 1265 in 2014). The frequency of SVT has increased marginally over the years (14.8% increase) relative to other arrhythmias, as shown in Figure 1 (*P* < .001 for all trends).

### 3.2 Demographics

**FIGURE 2:**
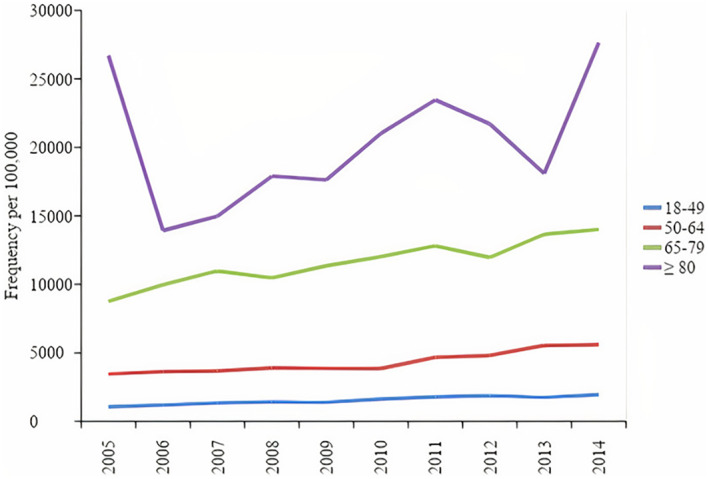
Temporal trends in frequency of any arrhythmia per 100 000 HIV hospitalizations within various age groups

Baseline characteristics of HIV‐related hospitalizations with arrhythmias are summarized in Table 2. Arrhythmias were most commonly present in patients aged 50 to 64 (49.3%). This is in contrast to the predominantly younger age group found in all hospitalizations. 61.2% of hospitalized HIV patients were in the 18 to 49 age range, and the 50 to 64 age group comprised 33.9% of all hospitalizations. While there was an increase in frequency of arrhythmias in all age groups, the increase in frequency was greatest in patients aged 18 to 49 (81% relative increase). Figure 2 illustrates the temporal trends in frequency of any arrhythmia per 100 000 HIV hospitalizations within various age groups.

**TABLE 2:** Baseline characteristics and course of hospitalization among hospitalized HIV patients from 2005 through 2014

	2005	2006	2007	2008	2009	2010	2011	2012	2013	2014	Overall	Relative change (%)	*P* value
Primary admission	4412	5629	5709	6197	6776	7873	8414	7955	8885	9435	71 285	108	<.0001
**Patient level variables**													
**Age**													
18‐49	1060	1190	1350	1430	1390	1630	1780	1870	1750	1940	1490	81	<.0001
50‐64	3460	3640	3680	3900	3870	3860	4680	4810	5550	5590	4370	64	<.0001
65‐79	8750	9980	10 980	10 470	11 360	12 020	12 820	11 970	13 660	14 010	11 970	53	<.0001
≥80	26 710	13 930	14 970	17 900	17 630	21 020	23 470	21 710	18 090	27 640	20 810	19	<.0001
**Gender**													
Male	2120	2530	2730	3050	3260	3410	4080	4190	4880	5080	3480	138	<.0001
Female	1310	1400	1690	1750	1650	2130	2490	2770	2780	3250	2090	147	<.0001
**Deyo/Charlson score**													
<2	1380	1620	1550	2060	1920	2050	2250	2070	2640	2740	2020	90	<.0001
≥2	2040	2330	2650	2800	3010	3290	4040	4370	4820	5170	3380	156	<.0001
**Comorbidities**													
Hyperlipidemia	3540	4350	4430	4990	5280	6750	6350	7430	8080	7930	6350	133	<.0001
History of hypertension	2960	3510	3850	4100	4360	4520	5480	5650	6180	6580	4830	119	<.0001
Diabetes mellitus	2530	3770	3920	4110	4440	4570	5670	6110	6550	7110	5000	165	<.0001
Chronic pulmonary disease	2400	3030	3220	3430	3280	3600	4460	5080	5760	5880	4070	144	<.0001
Peripheral vascular disease	5230	5010	7930	8040	6250	7980	9110	10 050	11 100	11 850	8820	123	<.0001
Neurological disorder or paralysis	1970	2410	2160	2670	2790	2890	3620	3470	4230	5140	3160	141	<.0001
Hematological or oncological malignancy	2520	2300	3320	3440	3260	4260	3980	4040	4090	4850	3660	86	<.0001
Anemia	1690	1990	2330	2780	2940	3210	4170	4320	4870	5400	3430	219	<.0001
Obesity	3080	3640	4700	4890	3980	5140	4380	5890	6630	6940	5310	108	<.0001
Coronary artery disease	7390	8120	8300	9210	9350	9630	11 090	11 660	12 080	13 110	10 330	75	<.0001
History of previous myocardial infarction	7050	5930	8140	10 290	7660	8350	11 630	10 860	10 860	12 570	9730	79	<.0001
History of previous CABG	9200	6720	6970	9440	11 930	11 750	14 650	14 220	14 850	15 180	12 210	96	<.0001
Congestive heart failure	9850	10 530	13 190	11 980	11 750	13 640	14 980	15 550	17 870	16 890	14 020	76	<.0001
Renal failure	4570	4690	5090	5500	5360	5960	7420	8030	8400	8870	6530	102	<.0001
**Hospital course**													
Use of vasopressor	10 640	9320	12 270	9640	16 270	15 110	13 180	14 770	17 710	16 590	14 180	69	<.0001
Cardiac catheterization	9310	10 480	11 400	12 110	13 430	12 940	10 780	14 250	14 150	16 050	12 590	56	<.0001
Endotracheal intubation	6840	6910	8780	8350	9680	9230	11 000	10 940	12 670	12 750	9650	89	<.0001
CPR	14 460	14 360	19 390	18 440	16 730	21 120	20 490	26 470	25 220	29 380	20 480	97	<.0001
Cardiogenic shock	28 090	22 160	25 100	28 000	34 320	34 470	36 550	35 400	30 100	37 600	32 900	42	<.0001
Cardiac arrest	14 020	15 370	18 960	18 620	20 220	20 510	21 620	25 520	26 140	28 650	21 060	97	<.0001
**Median household income category for patient's zip code**													
1. 0‐25th percentile	1840	2080	2170	2490	2490	2740	3430	3470	3850	4050	2800	124	<.0001
2. 26‐50th percentile	1740	2290	2360	2390	2940	3140	3600	3900	4300	4750	3050	165	<.0001
3. 51‐75th percentile	1890	2160	2540	3310	3470	3300	4530	4480	4830	5170	3440	177	<.0001
4. 76‐100th percentile	2350	2540	3130	3280	3570	4190	4760	4830	5640	5310	3790	144	<.0001
**Primary payer**													
Medicare/Medicaid	1900	2280	2550	2670	2780	3190	3750	3960	4470	4760	3200	149	<.0001
Private including HMO	2390	2190	2750	3160	3430	3370	3910	3950	4240	4140	3310	87	<.0001
Self‐pay/no charge/other	1020	1460	1290	1590	1760	1570	1830	2060	2360	2810	1710	143	<.0001
**Admission type**													
Nonelective	1910	2250	2460	2640	2730	3080	3610	3790	4270	4600	3090	138	<.0001
Elective	1550	1310	1630	2360	2610	2250	3030	3120	3360	3290	2380	138	<.0001
**Hospital characteristics**													
**Hospital bed size**													
Small	2110	1910	1900	2360	2230	2960	3170	3050	3540	4050	2770	98	<.0001
Medium	1330	2000	2470	2870	2800	3000	3160	3760	4260	4730	3030	221	<.0001
Large	2040	2230	2370	2560	2710	2980	3730	3800	4250	4430	3030	125	<.0001
**Discharge**													
Home	1670	1890	2040	2280	2370	2550	3080	3230	3650	3790	2620	130	<.0001
Facility	2350	2990	2930	3600	4100	4200	5260	5570	6610	7130	4420	201	<.0001
Length of stay during index admission in days (median, IQR)	6 (3‐11)	6 (3‐11)	5 (3‐10)	5 (3‐10)	5 (3‐9)	5 (3‐10)	5 (3‐9)	5 (2‐9)	5 (2‐9)	5 (3‐10)	5 (3‐10)		.0483
Cost of care (median, IQR)	12 285 (6446‐27 184)	12 974 (6502‐27 557)	12 181 (6117‐26 158)	11 486 (6107‐25 911)	12 362 (6029‐25 850)	12 029 (6210‐25 024)	12 570 (6396‐25 312)	12 043 (6274‐25 683)	11 602 (6111‐25 629)	12 410 (6410‐27 253)	12 210 (6248‐26 167)		.4019

Abbreviations: CABG, coronary artery bypass graft; CPR, cardiopulmonary resuscitation; HMO, Health Maintenance Organization; ICD, implantable cardioverter defibrillator; IQR, interquartile range.

The majority of arrhythmias were present in males (76.42%) compared to females (23.58%). Over the years, there was a comparable increase in the frequency of arrhythmias among both genders (138% in males and 147% in females). Arrhythmias were most frequent in patients within lowest quartile for household income (0‐25th percentile, 44.85%). Arrhythmias were less frequent with every increase in quartile for household income. Similar to all HIV hospitalizations, patients with arrhythmias were most commonly admitted nonelectively (90.77%). Patients with arrhythmia had a greater burden of comorbidities, as evidenced by a Deyo/Charlson score of ≥2, which was present in 81.64% of patients with any arrhythmia, compared to 72.42% found in patients with no arrhythmias (*P* < .0001).

**FIGURE 3:**
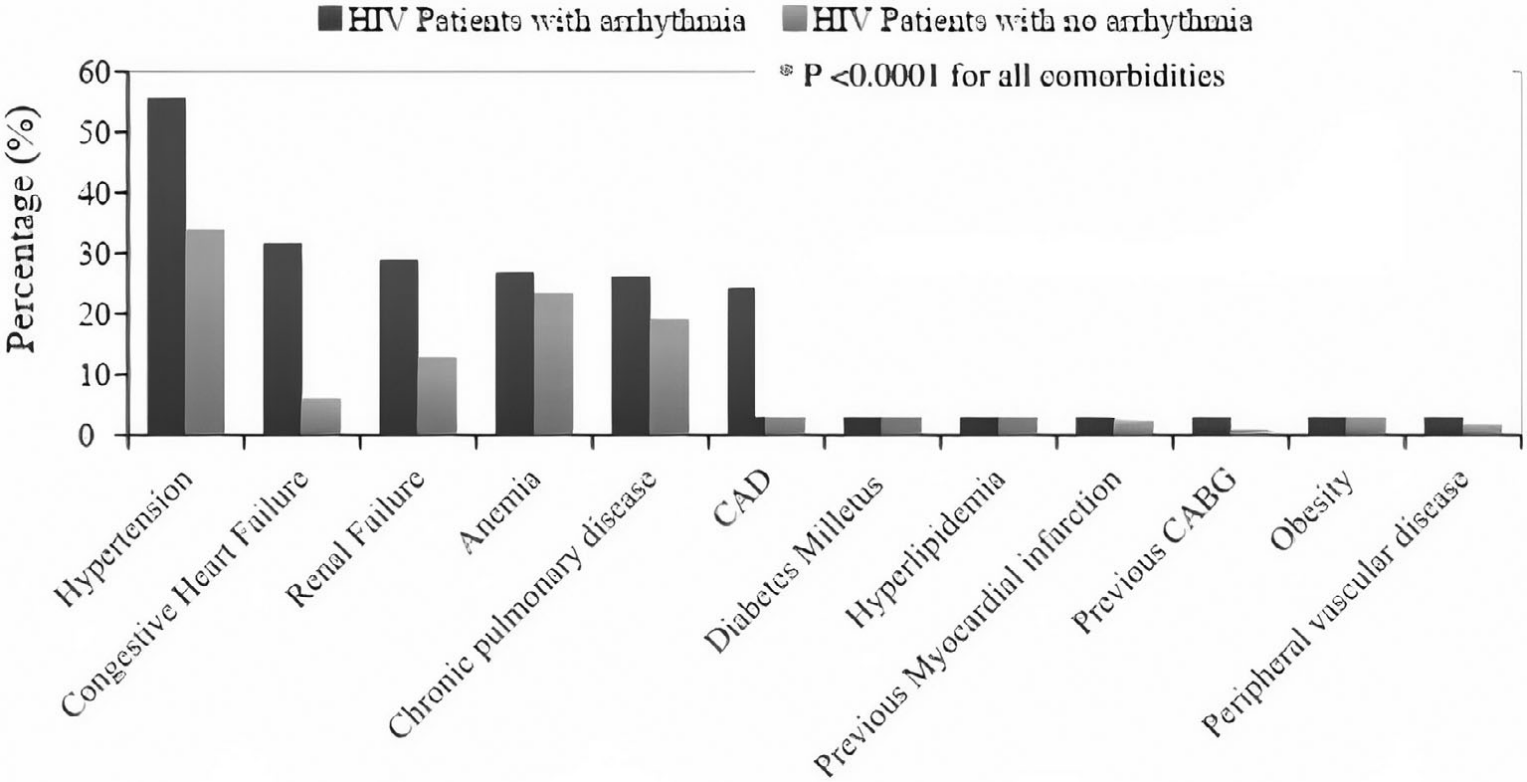
Percentage of comorbidities among HIV patients with arrhythmia and with no arrhythmia. CABG, coronary artery bypass graft; CAD, coronary artery disease

Among all comorbidities assessed, patients with any arrhythmia had a significantly greater frequency of hypertension (55.56% vs 33.94%), congestive heart failure (31.73% vs 6.03%), renal failure (29.06% vs 12.9%), chronic pulmonary disease (26.23% vs 19.16%), CAD (24.31% vs 6.54%), diabetes mellitus (23.43% vs 13.8%), hyperlipidemia (22.22% vs 10.16%), previous myocardial infarction (8.24% vs 2.37%), previous CABG (4.23% vs 0.94%) and obesity (6.98% vs 3.86%) compared to hospitalized patients with no arrhythmias (*P* < .0001 for all comorbidities) (Figure 3).

### 3.3 Hospital course in patients with any arrhythmia

Hospitalization course among patients with arrhythmias is summarized in Table 2. HIV‐related hospitalizations with arrhythmias had a statistically significant longer average length of stay, compared to without arrhythmias (5 days vs 4 days; *P* < .0001). The median cost of care was significantly higher in patients with arrhythmias compared to all hospitalizations ($12 210 vs $7665, IQR $6248‐$26 167 in patients with arrhythmias and $4435‐$14 336 in all hospitalizations; *P* < .0001). The median length of stay and cost of care in HIV‐related hospitalizations with arrhythmias has mostly remained unchanged over the years (*P* value of .0483 and .4019 respectively).

Markers of increased disease severity were more common among HIV‐related hospitalizations with arrhythmia compared to patients with no arrhythmia. These include vasopressor use (1.3% vs 0.24%), cardiac catheterization (7.17% vs 1.54%), cardiac arrests (4.09% vs 0.42%), CPR (3.4% vs 0.41%) and endotracheal intubation (12.75% vs 3.7%).

All‐cause in‐hospital mortality was associated with the presence of any cardiac arrhythmia. Patients with arrhythmias had an in‐hospital mortality rate of 9.6%, as opposed to a rate 2.84% found in patients with no arrhythmia. However, the in‐hospital mortality rate in patients with any arrhythmia has decreased over the years from 12.35% in 2005 to 7.9% in 2014 (decrease of 43.8%; *P* < .001 for trend). Among the arrhythmias, the highest reduction in in‐hospital mortality was observed in patients with VT (decrease of 57.5%; *P* < .0001 for trend).

As anticipated, the highest in‐hospital mortality was associated with patients who had VF (46.11%), followed by VT (14.48%), SVT (8.73%), and atrial flutter (7.51%). Despite being the most frequent arrhythmia in HIV patients, AF was associated with the lowest in‐hospital mortality (6.8%). Results for in‐hospital mortality throughout the years, stratified by arrhythmia type are summarized in Table 3.

**TABLE 3:** In‐hospital mortality in hospitalized HIV patients from 2005 through 2014, stratified by arrhythmia type

(%)	2005	2006	2007	2008	2009	2010	2011	2012	2013	2014	Overall	Relative change (%)	*P* value
Overall arrhythmia	12.35	11.21	12.97	10.7	10.21	10.31	9.19	7.48	7.32	7.9	9.6	−43.8	<.0001
Ventricular tachycardia	13.32	16.11	18.56	22.2	18.32	17.09	12.67	8.83	9.93	11.24	14.48	−57.5	<.0001
Ventricular fibrillation/flutter	56.58	50.89	57.93	49.57	47.67	46.64	42.28	39.39	42.19	34.72	46.11	−36.0	<.0001
Supraventricular tachycardia	9.77	4.22	7.68	10.38	10.42	9.33	11.64	9.72	5.49	7.58	8.73	1.2	<.0001
Atrial fibrillation	9.71	9.19	9.95	5.67	6.7	7.01	6.85	5.52	5.13	5.61	6.8	−47.0	<.0001
Atrial flutter	12.79	4.51	9.56	8.43	7.18	6.02	7.84	4.07	7.48	9.49	7.51	−16.8	<.0001

*Note*: Values are presented as percentages (%).

### 3.4 Association of arrhythmias in HIV patients with demographics and comorbidities

On multivariate analysis, later year of admission had a higher odds ratio (OR) for arrhythmia among HIV patients compared to earlier years (year 2014 vs 2005 OR was 1.391; *P* < .0001). Older age was also associated with arrhythmias (OR 1.055; *P* < .0001). Female sex was associated with lower odds of arrhythmias (OR 0.689, *P* < .0001).

Among comorbidities assessed, congestive heart failure had the strongest correlation with arrhythmias (OR 3.345; *P* < .0001), followed by obesity (OR 1.504; *P* < .0001), coronary artery disease (OR 1.46; *P* < .0001), renal failure (OR 1.397; *P* < .0001), hypertension (OR 1.205; *P* < .0001), and chronic pulmonary disease (OR 1.183; *P* < .0001).

In addition, cardiogenic shock, cardiac arrest and endotracheal intubation had high odds for arrhythmias (OR 3.18, 2.966, and 2.261, respectively; *P* < .0001).

Higher income was associated with arrhythmia‐related hospitalization in HIV patients (OR 1.279 in patients with top quartile for income; *P* < .0001). Other variables assessed were not strongly associated with arrhythmias. The multivariate predictors for arrhythmias are detailed in Table 4.

**TABLE 4:** Multivariate predictors of arrhythmias in hospitalized HIV patients

Variables	OR	LL	UL	*P*‐value
Year 2014 vs 2005	1.391	1.271	1.521	<.0001
**Age**	1.055	1.053	1.057	<.0001
**Female**	0.689	0.658	0.721	<.0001
**Comorbidities**				
Hyperlipidemia	1.116	1.06	1.175	<.0001
History of hypertension	1.205	1.155	1.257	<.0001
Diabetes mellitus	0.936	0.889	0.985	.0116
Chronic pulmonary disease	1.183	1.132	1.238	<.0001
Peripheral vascular disease	1.133	1.035	1.239	.0069
Neurological disorder or paralysis	0.988	0.928	1.053	.718
Hematological or oncological malignancy	1.088	1.006	1.177	.0339
Anemia	0.964	0.922	1.007	.0963
Obesity	1.504	1.391	1.627	<.0001
Coronary artery disease	1.46	1.375	1.549	<.0001
History of previous myocardial infarction	1.178	1.087	1.276	<.0001
History of previous CABG	1.099	0.985	1.225	.0909
Congestive heart failure	3.345	3.192	3.505	<.0001
Renal failure	1.397	1.331	1.465	<.0001
**Hospital course**				
Use of vasopressor	1.817	1.503	2.196	<.0001
Cardiac catheterization	1.748	1.602	1.907	<.0001
Endotracheal intubation	2.261	2.11	2.424	<.0001
CPR	1.916	1.642	2.234	<.0001
Cardiogenic shock	3.18	2.63	3.847	<.0001
Cardiac arrest	2.966	2.573	3.42	<.0001
**Median household income category for patient's zip code**				
0‐25th percentile	Referent	Referent	Referent	
26‐50th percentile	1.111	1.058	1.166	.0499
51‐75th percentile	1.233	1.171	1.299	.0002
76‐100th percentile	1.279	1.203	1.359	<.0001
**Primary payer**				
Medicare/medicaid	Referent	Referent	Referent	
Private including HMO	1.201	1.141	1.264	<.0001
Self pay/no charge/other	0.843	0.786	0.905	<.0001
Elective vs nonelective	0.808	0.757	0.862	<.0001
weekdays vs weekend	1.016	0.971	1.064	.4938
**Hospital bed size**				
Small	Referent	Referent	Referent	
Medium	1.115	1.038	1.198	.0261
Large	1.111	1.04	1.186	.021
Teaching vs nonteaching	0.971	0.932	1.011	.1489

Abbreviations: CABG, coronary artery bypass graft; CPR, cardiopulmonary resuscitation; HMO, Health Maintenance Organization.

## 4 DISCUSSION

In this analysis of HIV‐related hospitalizations from 2005 through 2014, we report an increase in the frequency of arrhythmias over time in this patient cohort. The presence of arrhythmia is associated with adverse outcomes, including a higher rate of in‐hospital mortality. In the current study, we report a 108% overall increase in the frequency of arrhythmia among HIV patients during the study period. To the best of our knowledge, this is the first study describing the temporal trends of arrhythmias in hospitalized HIV patients over time.

We found the following variables to be independently associated with the presence of arrhythmia in HIV‐related hospitalizations: male sex, older age, higher income, later year of admission and the presence of comorbidities such as congestive heart failure, obesity, coronary artery disease (CAD), renal failure, hypertension, chronic pulmonary disease, history of previous myocardial infarction, and peripheral vascular disease. It is notable that the frequency of these comorbidities has also increased over time, which could explain the rise in overall frequency of arrhythmias during the study period. Although the association between all‐cause in‐hospital mortality and arrhythmia was significant, the mortality rate among patients with any arrhythmia declined over the time. The contributory factors to this decrease are unclear, but could relate to improved diagnosis and management of cardiovascular disease. This trend was consistent with the decrement in all‐cause mortality found in HIV patients in general, as reported by multiple studies worldwide.^16^, ^17^, ^18^

AF was the most frequent arrhythmia among hospitalized HIV patients. Our reported frequency of AF was 2.11%. This is concordant with a previous analysis of the Veterans Affairs HIV Clinical Case Registry, which reported a frequency of 2.6% of atrial fibrillation in a large cohort of over 30 000 HIV patients from 1996 to 2011.^9^

While our study does not address the mechanisms underlying the significant frequency of arrhythmias in the setting of HIV, other studies provide insight. HIV infection is a known risk factor for atherosclerosis and stroke,^19^, ^20^ and similar mechanisms can explain the incidence of arrhythmias in that population. Both advanced age and inflammation have been associated with an increased risk for developing arrhythmias such as AF,^21^, ^22^ making older patients with chronic HIV particularly susceptible to developing it. Indeed, persistent immunodeficiency, accelerated immunosenescence and inflammation in HIV patients were found to accelerate the onset of age‐associated diseases, including cardiovascular diseases and arrhythmias such as AF.^23^ Elution of inflammatory cytokines and reactive oxygen species by infected cardiac endothelium; expression of HIV‐associated proteins that lead to destruction of mitochondria and myocardial damage are mechanisms previously implicated in the pathogenesis of AF in HIV infected patients.^24^ The severity of the HIV infection also correlates with risk of developing AF. A previous analysis identified low CD4+ cell count and high HIV RNA viral load as independent variables for the development of AF in HIV patients.^9^ The exact mechanism underlying this correlation is difficult to establish given that many patients in the HIV population share similar risk factors for AF, such as CAD and heart failure, as demonstrated by our study. Additionally, components of highly active antiretroviral therapy (HAART) such as protease inhibitors are associated with development of metabolic syndrome, which is a risk factor for AF.^25^

In our study, the frequency of malignant arrhythmias such as VF and VT has increased over the years. While the reason behind this rise is unclear, previous studies showed that patients with HIV can have a prolonged QTc, predisposing them to malignant arrhythmias including Torsades de Pointes, sustained VT and VF. This prolongation can be a direct result of the HIV infection itself, and can be observed in the absence of overt cardiovascular disease.^6^, ^26^ The severity of the HIV infection, evidenced by a low CD4+ cell count and high viral load, has been shown to be a risk factor for the development sudden cardiac death (SCD).^10^ Furthermore, medications frequently administered to HIV patients, including pentamidine, TMP‐SMX (Trimethoprime‐Sulfamethoxazole), non‐nucleoside reverse transcriptase inhibitor efavirenz (Sustiva), and protease inhibitors such as atazanavir (Reyataz),^27^ have also been associated with QTc prolongation.^6^, ^28^, ^29^ It is possible that the widespread use of such medications contributed to the rise of malignant arrhythmias in the HIV population over the years. The increased frequency CAD and CHF in the HIV population, as shown by our study, have likely contributed to the rise of such arrhythmias. These findings call for increased scrutiny to the QTc interval in HIV patients, and warrant considering HIV disease status in the risk assessment of malignant arrhythmias.

## 5 STUDY LIMITATIONS

Our study has a number of limitations. First, there are constraints with using administrative databases such as the NIS. It is a de‐identified database; making it impossible to validate individual ICD‐9 codes. Electrocardiograms and telemetry strips were not available to validate the type of arrhythmia in our sample. Furthermore, the use of ICD‐9 codes does not indicate whether the arrhythmia coded is new onset or existed prior to the ascertained hospitalization. The NIS also does not provide long term follow‐up data. It regards each hospitalization as a separate entity, so it is possible that readmissions were regarded as distinct hospitalizations, leading to an overestimation in the number of hospitalizations. The cause of in‐hospital mortality could not be ascertained since hospitalization notes are unavailable through the NIS. It is uncertain whether changes in coding practice patterns contribute to the changes in reported arrhythmia frequency, using NIS methodology. Furthermore, we cannot completely exclude the possibility that additional unknown confounding variables, not included in our analysis, may explain some of the associations found. We could not, for instance, ascertain the extent at which concurrent medication use such as anti‐retroviral therapy (ART) impacted the incidence of arrhythmias, nor could we determine the relationship between disease severity (CD4 count or viral load) and arrhythmia risk.

Future studies should be aimed at investigating the various drug regimens for HIV and their associated risk of developing arrhythmias. Data on CD4+ cell count and HIV viral load was not collected in our sample as they were not available through the NIS. Lastly, our sample did not include uninfected controls to directly compare the burden of arrhythmias between HIV and non‐HIV patients. Unfortunately this comparison was not feasible to perform due to the very large number of uninfected controls available through the NIS registry. Such a study is warranted to determine if HIV infection is an independent risk factor for the development of arrhythmias. Unfortunately, it was not possible to perform this analysis using the NIS registry. These limitations are, however, counterbalanced by the presence of a large unrestricted study sample which lacks the selection bias found in studies reported by individual specialized centers and skilled operators.

## 6 CONCLUSION

Among hospitalized HIV patients, cardiac arrhythmias are associated with significant morbidity and mortality. AF is the most frequent arrhythmia among hospitalized HIV patients. The presence of arrhythmias is associated with adverse outcomes in HIV patients, including a higher in‐hospital mortality rate and cost of care. The in‐hospital mortality among patients with any arrhythmia is significant but has decreased over the years.

## CONFLICT OF INTEREST

The authors declare no potential conflict of interests.

## Data Availability

The following information was supplied regarding data availability: The NIS database raw files were purchased through online HCUP (health care cost and utilization project) distributor. All purchasers and users of HCUP data must complete the online Data Use Agreement (DUA) training so that they are familiar with the rules and restrictions for the use of HCUP data. If this raw data is made public, then the likelihood of maintaining standards to complete the DUA training might be violated. Moreover, anyone who uses the database files we purchased must complete an HCUP Data Use Agreement form (a form which the owner of the data has to generate and acknowledge). Therefore, given these limitations, we have provided raw data for review purposes and not for publishing (i.e., to be shared with the public). Additionally, as per AHRQ‐HCUP, before the reviewers/editors may access any HCUP data (the raw files), they are required to complete the 15‐minute Data Use Agreement (DUA) training and read and sign the HCUP DUA. Both of these may be completed online at this link: https://www.hcup-us.ahrq.gov/tech_assist/dua.jsp
